# Ligation of the Vertebral Arteries in Epilepsy

**Published:** 1886-09

**Authors:** 


LIGATION OF THE VERTEBRAL ARTERIES IN EPILEPSY.

In another part of this issue is a brief abstract of a paper on the above subject, read by Dr. J. L. Gray before the Chicago Medical Society. Several points raised in that article are of considerable interest. The theory of Alexander (*Brain, 1882*) is, in brief, that the operation was of benefit in modifying the circulation by diminishing the quantity of blood sent to the diseased and hyper-sensitive centers. Dr. Gray, in his paper, says that he regards the very free anastomosis existing in the circle of Willis as sufficient to re-establish the circulation in a short time, while Alexander would lead us to think that the complete dilatation of the posterior communicating arteries may require months or even years. In support of his views, Dr. Gray pertinently observes that no evidence of anæmia is observable in the parts supplied by the arteries, immediately after the operation; and further, that were the benefit entirely due to the impression made upon the circulation, it would be as great in those cases in which the excitation starts in the cortex and is propagated downward as in those in which it originates in some region outside of the central nervous system; but this does not seem to be the case.

Professor Jewell regards epilepsy as essentially a disease of the vaso-motor nerves. He has recommended the tying of the vertebrals, on the ground that the vaso-motor nerves of the co-ordinating center of the brain, which accompany the arteries and are intimately blended with their coats, are thereby cut off. Theoretically speaking, in proportion to the degree to which the vaso-motor nerve-supply of the convulsive centers is cut off, ought the operation to be successful. Guided by these considerations, he has recommended that the operations be done as high as practicable.

In Alexander's operations the artery was ligated at the sixth cervical vertebra. He reports twenty-one cases, three of which have remained well for upward of a year. Nine cases bid fair to be classed as recoveries, though sufficient time has not yet elapsed. Eight were greatly improved.

The operation has been performed, in this city seven times, with one death. Of the other six cases, three are reported, though in not very distinct terms, as having been benefited. One received no benefit (cortical epilepsy), and two are so recent that nothing can yet be affirmed as to the result. In all these cases the artery was ligated between the atlas and the axis.

It would seem, from an analysis of the above results, that the so-called high operation has little to recommend it over that first proposed by Dr. Alexander. But as yet it is credited with too few cases, and such a short time has elapsed since it was recommended, that we are hardly justified in coming to any conclusions as between the two. It may prove necessary to sever the communication between the superior and middle sympathetic cervical ganglia—theoretically it should—practically it seems to make little difference.

Ross states that obstruction of the basilar artery at its inferior extremity causes paralysis of all four extremities and both sides of the face. Simultaneous obstruction of both vertebrals produces exactly the same effect, while in obliteration of one vertebral the symptoms assume a hemiplegic form.

It may readily occur, in obstruction dependent on arterial disease, that the collateral circulation is with difficulty established, and softening and paralysis take place. But just how an embolism—say from the aortic valves—in occluding an artery could cause different symptoms from a ligature applied in the same situation, is not apparent.

We need more light on this subject, and it would seem a fruitful field for experimental pathologists.

